# Secondary Parkinsonism in a Patient With a Cerebral Cavernous Hemangioma Treated With Stereotactic Radiosurgery

**DOI:** 10.7759/cureus.14128

**Published:** 2021-03-26

**Authors:** Seckin Aydin, Aysegul Esen Aydin, Odhan Yuksel, Taner Tanriverdi

**Affiliations:** 1 Department of Neurosurgery, Prof. Dr. Cemil Tascioglu City Hospital, University of Health Sciences, Istanbul, TUR; 2 Department of Neurosurgery, Bakirkoy Research and Training Hospital for Neurology, Neurosurgery and Psychiatry, Istanbul, TUR; 3 Department of Neurosurgery, Baskent University School Medicine, Alanya Teaching and Medical Research Center, Alanya, TUR; 4 Department of Neurosurgery, Cerrahpasa Medical Faculty, Istanbul University-Cerrahpasa, İstanbul, TUR

**Keywords:** cerebral cavernous hemangioma, secondary parkinsonism, stereotactic radiosurgery (cyberknife®)

## Abstract

Secondary parkinsonism is defined with some symptoms similar to idiopathic Parkinson's disease, but with different etiologies. And cerebral cavernous hemangioma is one of the rare cases. A 51-year-old, male patient was consulted with tremor, rigidity and bradykinesia on the right upper extremity. The Hoehn and Yahr Parkinson’s scale was Stage І. Radiological evaluations showed a deep-seated cerebral cavernous hemangioma at the left posterior insular region. The patient received stereotactic radiosurgery (CyberKnife®, Accuray Incorporated, Sunnyvale, CA, USA). Clinical and radiological improvements revealed within follow-up, respectively. Stereotactic radiosurgery may be an alternative treatment for secondary parkinsonism by reducing the risk of re-bleeding and reducing its size.

## Introduction

Parkinsonism is a clinical syndrome characterized by some cardinal findings related to Idiopathic Parkinson’s disease including bradykinesia, tremor, rigidity and postural instability [1]. Secondary parkinsonism resembles Idiopathic Parkinson’s disease clinically, but etiology is different in secondary parkinsonism including drugs, trauma, normal pressure hydrocephalus, toxins or vascular pathologies [2]. Secondary parkinsonism constitutes approximately 14%-16% of all parkinsonism cases over 40 years of age [3]. The absence of asymmetric symptoms, resting tremor and unresponsiveness to levodopa (L-dopa) treatment at the beginning of the disease differentiates it from Idiopathic Parkinson’s disease. In the literature, there have been reported some cases that cerebral cavernous hemangioma (CCH) may be a cause of secondary parkinsonism.

In recent years, stereotactic radiosurgery (SRS) is more often performed with wider indications and experience is also increased consequently. It becomes an alternative treatment option for symptomatic deep-seated CCH that carries high operative risk potentially and also, for patients who refuse surgery [4]. In accordance with this manner, we reported a CCH case treated with SRS located in the left posterior insular region caused secondary parkinsonism. Additionally, we reviewed the limited number of similar cases reported in the literature so far. This study has been conducted in accordance with the Declaration of Helsinki, and an informed consent form was obtained from the patient.

## Case presentation

**Figure 1:**
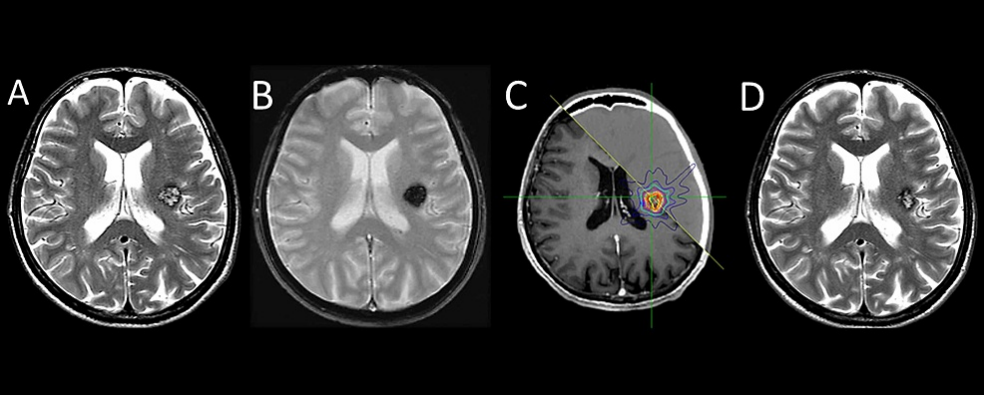
Radiological imaging MRI axial plane, T2-weighted image shows a cavernous hemangioma with peripheral hemosiderin rim at the left posterior insular region (A), MRI axial plane, gradient echo image shows hemorrhagic lesion (B), a fusion of axial planes of MRI and computed tomography shows the target delineation for cavernous hemangioma with isodose distribution (C), MRI axial plane, T2-weighted image shows a considerable decrease in the size of cavernous hemangioma in 30 months follow-up after CyberKnife (D).

A 51-year-old male was admitted to our clinic with a right arm tremor that started six months ago and increased in amplitude one month before the admission. There was no chronic disease in his medical history and laboratory screening showed nothing abnormal. Neurological examination showed parkinsonian tremor with the right hand, bradykinesia, rigidity, mild right upper extremity motor dysfunction and clumsiness. The Hoehn and Yahr Parkinson’s scale was Stage І. Magnetic resonance imaging (MRI) revealed CCH located on the left posterior insular region with 16x15x15 mm in size. Classical hemosiderin rim was noted on T2-weighted image with hypointensity (Figure 1A). Gradient echo image demonstrated the pathognomonic sign (Figure 1B). L-dopa treatment was started to treat parkinsonian symptoms. However, the patient did not give a response to L-dopa treatment. Firstly, L-dopa dose was increased. Thereafter, multiple medical treatments were initiated because of persistent symptoms and end-of-dose motor fluctuations. Because of the deep-seated location of CCH, surgical evaluation was not performed and the patient underwent SRS CyberKnife® (Accuray Incorporated, Sunnyvale, CA, USA) with 16 Gy dose in a single fraction (Figure 1C). The patient continued multiple medical treatments and was followed-up by the neurology department. Radiological evaluations were performed at three-month intervals during the first year and then six-monthly. Symptoms were not worsened in long term follow-up of 54 months, and the Hoehn and Yahr Parkinson’s scale was still Stage І. Also, a considerable decrease in the size of the CCH developed in follow-up (Figure 1D).

## Discussion

CCH is a vascular lesion with irregular thin walls without intervening cerebral tissue and capillary. Sinusoidal and cavernous types have been reported so far [5]. Terminologically, it is also described as cavernoma, cavernous angioma, venous vascular malformation or vascular hamartoma.

The prevalence of CCH is 0.5% and it constitutes 5%-13% of all cerebrovascular malformations. Seizure and bleeding constitute the most common neurological symptoms. The natural history of CCH reveals a “temporal clustering’’ pattern describing long bleeding-free intervals in between symptomatic episodes. The emerging clinical symptoms due to bleeding usually tend to be improved by the resorption of the blood, unless there is a permanent injury in the healthy tissues. They are mostly located in the supratentorial areas. Deep-seated CCHs are not common, and the location at the basal ganglia has been reported to be only 6%-8% [5,6]. Developing secondary parkinsonism is exceptional during the clinical course of this pathology. The annual bleeding rate is around 0.1%-2.5% per lesion and 0.25%-16.5% per patient [7]. The history of prior bleedings increases the risk and, when the bleeding is confirmed twice by both clinical and radiological findings, then, the risk is reported to be at least 34% per year [8].

MRI is the gold standard for the diagnosis of CCH. The lesion is usually characterized by a central hypointensity in a reticular form on T2-weighted images and a peripheral hypointense ring due to the collection of hemosiderin. Gradient echo images are highly sensitive and make the diagnosis easier. Zabramski et al. classified CCH’s into four groups based on their radiographic features [9]. Our case belongs to type 2 depending on this classification.

The efficiency of SRS has long been known for the treatment of central nervous system primary tumors and metastases [10]. SRS can also be utilized to treat movement disorders [11]. The effect mechanism is to constitute focal lesions with thalamotomy or pallidotomy. Successful treatment results were obtained in patients who are not suitable for open surgery. In the literature, there are studies reporting that intractable tremor improved over 90% in patients who underwent thalamotomy with SRS [12]. SRS has also been performed for vascular brain pathologies for a long time. It is known that SRS causes progressive endothelial cell proliferation and luminal closure on arteriovenous malformation [13]. Some authors claimed that SRS treats CCHs by a similar mechanism [14,15].

Treatment of CCH is still controversial. Due to a lower risk of bleeding, incidentally diagnosed CCHs are treated with conservative modalities. On the other hand, symptomatic and easily accessible CCHs can be operated with microsurgery. If deep-seated lesions reach the pial surface and the bleeding results in progressive neurological deterioration, surgical intervention will be required [16]. A similar approach is also considered to be appropriate for cases with intractable epilepsy [17]. It has been argued that SRS may be a good alternative in selected patients with deep-localized CCH, who are considered to be at high risk of surgery [6]. When SRS was performed in these patients, the risk of re-bleeding was demonstrated to be decreased significantly [18]. Also, it has been reported that CCH patients treated with SRS have a regression of the size in follow-up periods longer than two years [6].

On the other hand, where CCHs are treated conservatively within long follow-up periods, decreasing the size may occur due to the nature of the CCH [19]. It is not clear whether SRS or the nature of the CCH is effective in ameliorating the patient's symptoms. In our case, no clinical or radiological findings to consider re-bleeding were identified during a follow-up period of 54 months. Furthermore, regression in the lesion was observed in the most recent MRI examination.

## Conclusions

We consider that the CCH in the cortico-striato-thalamic pathway might potentially have exerted pressure or caused bleeding leading to the parkinsonism symptoms of the patient. SRS has started to gain importance in the treatment of many neurovascular and neuro-oncological brain pathologies with an expanding application field. In this report, we would like to highlight for the first time that SRS might be an alternative treatment of this rare entity. 
